# Synergistic effects of combined exposure to hydroquinone and nitrofurantoin in T24 bladder cells: an *in vitro* toxicology study with implications for risk assessment

**DOI:** 10.2478/aiht-2026-77-4146

**Published:** 2026-09-25

**Authors:** Ana Huđek Turković, Željka Stanečić, Ana Butorac, Vilena Kašuba, Marijana Ćurčić, Antonio Gagić, Ivana Šola, Marija Lovrić, Gordana Rusak, Ksenija Durgo

**Affiliations:** University of Zagreb Faculty of Food Technology and Biotechnology, Zagreb, Croatia; BICRO BIOCentre Ltd., Central Laboratory, Zagreb, Croatia; Institute for Medical Research and Occupational Health, Zagreb, Croatia; University of Belgrade Faculty of Pharmacy, Department of Toxicology “Akademik Danilo Soldatović”, Belgrade, Serbia; University of Zagreb Faculty of Science, Department of Biology, Zagreb, Croatia; Selvita Ltd., Zagreb, Croatia

**Keywords:** apoptosis, CBMN cytome assay, cytotoxicity, DNA damage, genotoxicity, oxidative stress, proteomics, tandem mass tag labelling, apoptoza, citomski mikronukleus (MN) test, citotoksičnost, genotoksičnost, oksidacijski stres, oštećenje DNA, proteomika, tandemsko označavanje masenim oznakama

## Abstract

Hydroquinone, the main active component of bearberry [*Arctostaphylos uva-ursi* (L.) Spreng.], is sometimes used together with the antibiotic nitrofurantoin to treat urinary tract infections. The aim of this *in vitro* study was to test the hypothesis that co-exposure to hydroquinone and nitrofurantoin would result in greater cytotoxic, oxidative, and genotoxic effects than exposure to either compound alone. To do that, we assessed single and combined toxic effects of two- and eight-hour exposure to both compounds in T24 human bladder carcinoma cells due to their stability and retention of key signalling pathways involved in oxidative stress, DNA repair, and apoptosis. Cytotoxicity and oxidative stress were assessed with the neutral red and 2′,7′-dichlorodihydrofluorescein diacetate assays and genotoxicity with the cytokinesis-block micronucleus cytome assay. Proteomic changes were examined using tandem mass tag labelling followed by mass spectrometry. Combined exposure to hydroquinone and nitrofurantoin produced a synergistic increase in cytotoxic and genotoxic effects compared to single-compound treatments, including reduced nuclear division index and activation of pathways related to oxidative stress, DNA damage, and proteotoxic stress. These findings highlight the importance of evaluating interactions between plant-derived hydroquinone and nitrofurantoin in risk assessment of combined exposure during therapy.

## Abstract (hr)

Hidrokinon, glavna aktivna komponenta medvjetke [*Arctostaphylos uva-ursi* (L.) Spreng.], ponekad se koristi zajedno s antibiotikom nitrofurantoinom za liječenje infekcija mokraćnog sustava. Cilj ove *in vitro* studije bio je testirati hipotezu hoće li istodobna izloženost hidrokinonu i nitrofurantoinu rezultirati većim citotoksičnim, oksidacijskim i genotoksičnim učincima, nego izloženost bilo kojem spoju zasebno. U tu svrhu procijenili smo pojedinačne i kombinirane toksične učinke dvosatne i osmosatne izloženosti obaju spojeva u stanicama humanoga karcinoma mjehura T24 zbog njihove stabilnosti i zadržavanja ključnih signalnih putova uključenih u oksidacijski stres, popravak DNA i apoptozu. Citotoksičnost i oksidacijski stres procijenjeni su Neutral Red i 2′,7′-diklorodihidrofluorescein diacetatnim testom, a genotoksičnost mikronukleus-testom s blokiranom citokinezom (tzv. citomska inačica testa). Proteomske promjene istražene su korištenjem tandemskog označavanja masenim oznakama te masenom spektrometrijom. Kombinirana izloženost hidrokinonu i nitrofurantoinu proizvela je sinergističko povećanje citotoksičnih i genotoksičnih učinaka u usporedbi s tretmanima jednim spojem, uključujući smanjeni indeks diobe jezgre i aktivaciju putova povezanih s oksidacijskim stresom, oštećenjem DNA i proteotoksičnim stresom. Ovi rezultati ističu važnost procjene interakcija između hidrokinona biljnoga podrijetla i nitrofurantoina u procjeni rizika kombinirane izloženosti tijekom terapije.

In recent years, the use of phytotherapeutic products, including plant extracts, herbal preparations, and homeopathic remedies, has increased substantially (1). Although often perceived as inherently safe, herbal products may have toxic effects or alter the therapeutic efficacy and safety of pharmaceuticals (2). Several studies suggest that combining natural compounds with antibiotics may enhance antimicrobial efficacy (3), but the potential toxicological consequences of such combinations on non-target human cells are still poorly characterised.

One combination that has seen a rise in the treatment of urinary tract infections involves the supplementation of nitrofurantoin, a broad-spectrum antibiotic, with remedies based on *Arctostaphylos uva-ursi* (L.) Spreng. (bearberry), a traditional medicinal herb with urinary antiseptic properties attributed to hydroquinone released from the hydrolysis of arbutin, the predominant bioactive compound in its leaves (4). However, this combination has scarcely been evaluated for potential interactions and toxic effects that could inform risk assessment. Our study aimed to address this gap by investigating combined cytotoxic and genotoxic effects of hydroquinone and nitrofurantoin *in vitro.* The fact that both compounds are extensively metabolised and excreted via urine makes the bladder epithelium one of the primary tissues exposed to their potential toxic effects. Therefore, we selected the T24 human bladder cell line as an appropriate model, which constitutes mechanistically relevant and ethically acceptable tool for toxicological evaluation in accordance with the 3R toxicological principles. This cell line retains functional pathways involved in oxidative stress regulation, DNA repair, and apoptosis, which are central to the evaluation of cytotoxicity and genotoxicity (5, 6). In addition, their well-characterised and stable phenotype ensures experimental reproducibility, which is particularly important when studying unknown synergistic effects. Finally, the sensitivity of T24 cells to redox-active agents facilitates the detection of subtle proteomic and genomic alterations, allowing for a detailed mechanistic understanding prior to confirmatory studies in non-tumorigenic models. Thus, the use of T24 cells provides an efficient and biologically relevant platform for elucidating the molecular basis of combined toxicity and potential oxidative synergy between hydroquinone and nitrofurantoin.

## MATERIALS AND METHODS

### Cell culture and drug treatment

The T24 human urinary bladder carcinoma cell line (ATCC HTB–4), kindly provided by the University of Split Faculty of Chemistry and Technology (Split, Croatia), was selected because of its uroepithelial origin and because both nitrofurantoin and hydroquinone are excreted in urine and are therefore in direct contact with the bladder epithelium. Even though the T24 cell line is neoplastic, it retains key signalling pathways related to oxidative stress, DNA damage response, and apoptosis, and is therefore suitable for mechanistic studies of cytotoxicity, genotoxicity, and proteomic alterations. Furthermore, its genetic and phenotypic stability allows for reproducible assessment of synergistic interactions between compounds.

The cells were cultured in Ham's F-12 with L-glutamine medium supplemented with 10 % foetal bovine serum (FBS) and 1 % antibiotics (penicillin 1000 U/mL, streptomycin 100 mg/mL) and kept at 37 °C in a humidified atmosphere with 5 % CO_2_ (Brouwer CH, Luzern, Switzerland). All experiments were conducted on the T24 cells between passages 5 and 15 during their logarithmic growth phase. The cells were treated with nitrofurantoin, hydroquinone, and their mixtures for 8 h (Table 1), which corresponds to the average overnight exposure during sleep. An equal amount of drug-free DMSO was used as negative control, considering that the initial solution of nitrofurantoin was prepared in DMSO.

**Table 1:** Substances and their concentrations used in experiments

**Name**	**Concentration (μg/mL)**	**Description**
Nitrofurantoin^*^	167 (0.16 % DMSO)	prophylactic dose (100 mg of nitrofurantoin consumed orally)
	330 (0.3 % DMSO)	minimal therapeutic dose (50 mg, 4× daily – 200 mg/day)
	670 (0.6 % DMSO)	maximal therapeutic dose (100 mg, 4× daily – 400 mg/day)

Hydroquinone	5; 20; 50; 320; 900	concentrations that are present in the urinary bladder after standard therapeutic application of bearberry extract (15)

* All concentrations are expressed if only 50 % of nitrofurantoin is eliminated through urine (300 mL); conversion factor 1.66 (33)

### Chemicals and reagents

Analytical standard of hydroquinone, neutral red dye, 2′,7′–dichlorofluorescein diacetate (DCFH-DA), formic acid, H_2_O_2_, cytochalasin B, and doxorubicin were purchased from Sigma Aldrich (St. Louis, MO, USA). Ham's F–12 with L-glutamine medium and FBS were obtained from Capricorn Scientific (Ebsdorfergrund, Germany). Nitrofurantoin was procured from Tokyo Chemical Industry Co., Ltd. (Chennai, India). DMSO and ethanol were purchased from Kemika (Zagreb, Croatia). Triethyl ammonium bicarbonate (TEAB) and tandem mass tag (TMT) duplex kit were procured from Thermo Fisher Scientific (Waltham, MA, USA). Acetonitrile was procured from VWR Chemicals (Radnor, PA, USA). Giemsa stain and glacial acetic acid (≥99 %) were obtained from Merck (Darmstadt, Germany).

The concentrations of hydroquinone used in this study are based on concentrations determined in bearberry [*Arctostaphylos uva-ursi* (L.) Spreng.] extract in our previous study (8). Two concentrations (320 and 900 μg/mL) showed toxic effect and two (20 μg/mL and 50 μg/mL) represent potential therapeutic levels after *in vitro* digestion of the *Uva-ursi* extract. Furthermore, we needed to reduce the therapeutic concentrations for the cytokinesis-block micronucleus (CBMN) cytome assay to 5 and 10 μg/mL, as explained below.

The nitrofurantoin concentrations were selected based on the recommended dosing regimens (8), with 330 and 670 μg/mL corresponding to the lowest and highest therapeutic doses, respectively, whereas the 167 μg/mL concentration corresponds to the prophylactic oral dose of 100 mg.

### Neutral red cytotoxicity assay

Cells were seeded in 96-well plates at a concentration of 10^5^ cells/mL and grown for 24 h before the 8-hour treatment. After the treatment, they were washed twice with phosphate-buffered saline (PBS). The cytotoxic effect was determined using the neutral red assay following the protocol described by Babich and Borenfreund (9). Absorbance was measured spectrophotometrically at 540 nm using a Fluostar OPTIMA plate reader (BMG Labtech, Durham, NC, USA). We conducted the experiment using two different approaches: one without recovery, where cell survival was measured immediately after treatment, and the other with recovery, where the medium was added and cell survival measured 24 h after the treatment. The percentage of surviving cells was calculated in comparison to the negative control (100 %).

### Reactive oxygen species determination

Cells were seeded as described above and the DCFH-DA fluorometric assay used to measure the amount of reactive oxygen species (ROS) with and without 24-hour recovery after treatment as described by Silveira et al. (10). Fluorescence intensity was measured at an excitation wavelength of 485 nm and an emission wavelength of 520 nm. The results are expressed as increase or decrease in fluorescence intensity relative to negative control. Cells treated with 10 % H_2_O_2_ for 15 min served as positive control.

### Cytokinesis-block micronucleus cytome (CBMN Cyt) assay

T24 cells (1×10^4^ cells/mL) were seeded in 24-well plastic plates with 14 mm diameter oval glass coverslips (Paul Marienfeld GmbH, Lauda-Königshofen, Germany) containing complete medium described above. Cells were treated for two and eight hours with nitrofurantoin (167 μg/mL), hydroquinone (5 or 10 μg/mL), or one of their combinations. For positive control we used doxorubicin (0.4 μg/mL), while the negative control was treated with 0.16 % v/v DMSO. The CBMN cytome assay was performed as described by Savio et al. (11), following the OECD Guideline No. 487 (12). Cytochalasin B was used at 3 μg/mL for 24 h. After processing cell cultures, prepared slides were dried and refrigerated at 20 °C until microscopy in oil immersion (1000×magnification; Olympus CX41, Tokyo, Japan). The frequencies of micronuclei (MN), nuclear buds (NB), and nucleoplasmic bridges (NPB) were measured in six thousand binucleated cells as described by Fenech and Crott (13), while the mitotic index and apoptotic and necrotic rates were measured as described by Eastmond and Tucker (14) in thousand cells per each experimental point.

### Protein analysis

The cells were seeded in 6-cm cell culture plates at a density of 1×10^6^ cells per plate and grown for 24 h under conditions described above. After incubation, the cells were treated with nitrofurantoin (167 μg/mL) or hydroquinone (20 μg/mL) or their mixture for 8 h and then rinsed with PBS, resuspended in lysis buffer (1 % sodium dodecyl sulphate in 100 mmol/L TEAB), and sonicated on ice using a Sonopuls HD 3200 homogeniser (Bandelin, Berlin, Germany) in three cycles at 10 s amplitudes of 30 %, separated by 20 s cooling pauses, as described in the user manual (15).

The obtained suspension was then centrifuged at 10000×*g* and 4 °C for 10 min and the supernatant analysed for total protein concentration using the Bradford assay (16).

Reduction, alkylation, digestion, and TMT labelling followed the manufacturer's instructions (15). Three biological replicas were analysed per group.

Peptide separation and mass spectrometry were performed using the SCIEX TripleTOF 6600+ system equipped with an Optiflow Turbo V Ion Source (Sciex, Framingham, MA, USA). Chromatographic separation was performed on a Phenomenex (Torrance, CA, USA) Luna Omega Polar C18 column (100 A, 150×0.3 mm) at 27 °C. The flow rate was set to 5 μL/min. Mobile phases A and B consisted of 0.1 % formic acid in water and 0.1 % formic acid in acetonitrile, respectively. Gradient elution was set as follows: 5–30 % B (0–68 min), 30–40 % B (68–73 min), 80 % B (73–78 min), 80–3 % B (78–79 min), and 3 % B (79–87 min). The ionisation source operated in positive ion mode with the following parameters: ion spray voltage of 4500 V, nebuliser gas 1 at 30 psi, nebuliser gas 2 at 35 psi, and curtain gas at 30 psi. The mass spectrometer scanned full spectra (*m/z* 400–1500) for 250 ms. Top 50 precursors were selected for MS/MS fragmentation (*m/z* 100–2000) with a charge state between +2 and +5 and counts above a minimum threshold (100 counts per second). Rolling collision was employed with the energy spread of 5. After MS acquisition, the obtained MS/MS spectra were used for database search using the ProteinPilot Software 5.0.2 (AB Sciex, Marlborough, MA, USA). The *Homo sapiens* proteome database was downloaded from the UniProt database (entry number 26610; revised SwissProt sequences; accessed on 1 August 2024). Search parameters included one missed trypsin cleavage, carbamidomethylation, and TMT6plex as fixed modifications. Precursor ion and fragment ion mass tolerance were set to 0.05 and 0.10 Da, respectively. The results were refined to include only proteins meeting the criterion of having a minimum of two identified peptide sequences and a false discovery rate of less than 1 %.

Enrichment analysis was performed on sets of genes that were up-regulated or down-regulated under the studied conditions using the Gene Ontology (GO) PANTHER 18.0 engine (University of Southern California, Los Angeles, CA, USA) which employs Fisher's exact test and false discovery rate correction. Overlapping proteins between the conditions were illustrated with the Venn diagram (17).

### Statistical analysis

Dose-response [benchmark dose (BMD) modelling] of the single hydroquinone (0–900 μg/mL) and single nitrofurantoin concentrations (0–670 μg/mL) and their mixtures was analysed on the PROAST software package (RIVM, Bilthoven, The Netherlands) as described by More et al. (18).

The effect of mixtures in relation to individual compounds was analysed using the factorial regression model within the STATISTICA software package version 7.0 (StatSoft, Tulsa, OK, USA) according to the following equation:

[1] $$\text{y}=\beta_{0}+\beta_{1}{\text{x}}_{1}+\beta_{2}{\text{x}}_{2}+\beta_{12}{\text{x}}_{1}{\text{x}}_{2}\ldots +ƭ$$

where y represents the effect, *β* the unknown parameters, *x_1_* hydroquinone, and *x_2_* nitrofurantoin. If *β*_12_ is negative, the chemicals within the mixture act antagonistically. If *β*_12_ is positive, the effect is considered synergistic.

To evaluate treatment differences in neutral red and DCHF-DA assays, we ran one-way ANOVA and post-hoc Tukey's HSD test. The Kolmogorov-Smirnov test was used to evaluate the distribution of data for normality and the chi-squared test to compare the results of the CBMN cytome test.

Statistical analysis of proteomics data was performed using the Scaffold Q+S program 5.3.0 (Proteome Software, Portland, OR, USA). Quantification was based on the reporter ion signal employing ratio-based normalisation and Permutation tests.

Statistical significance for all tests was set to P<0.05.

## RESULTS AND DISCUSSION

### Cytotoxicity and ROS findings

**Figure 1:**
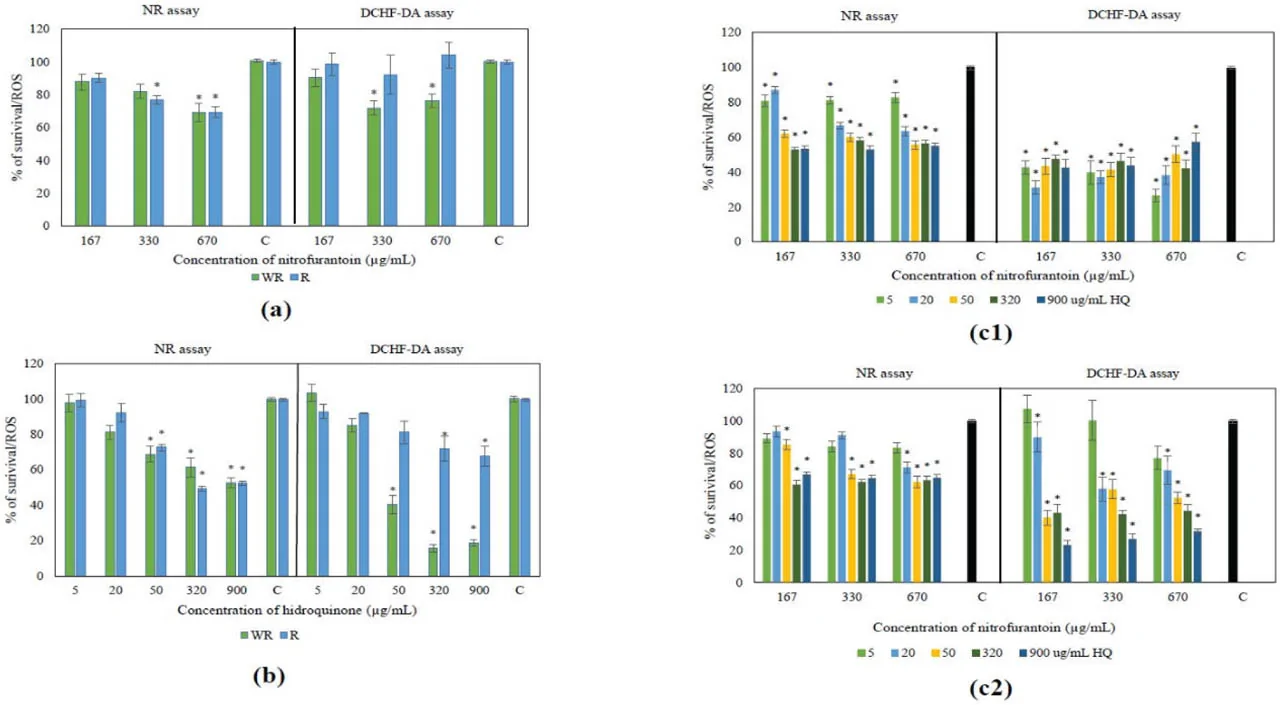
Cytotoxic (neutral red) and antioxidative/prooxidative (DCHF-DA) activity of a) nitrofurantoin, b) hydroquinone, and their mixture in unrecovered (c1) and recovered over 24 h (c2) human urinary bladder cancer T24 cells. * Statistically significant result compared to the control (100 %; one-way ANOVA followed by post hoc Tukey's HSD test; P<0.05)

Compared to control, the highest therapeutic dose of nitrofurantoin (670 μg/mL) significantly reduced T24 cell survival to below 80 % after 8 h of treatment (P<0.05; Figure 1a). Similar effects were observed after the 24-hour recovery period, suggesting that the damage at the cellular level is permanent. Both therapeutic doses exhibited lower antioxidative activity in unrecovered T24 cells, possibly contributing to their lower survival.

DMSO reduced T24 cell survival by 1.5 %, and all DMSO concentrations in nitrofurantoin samples (0.16–0.6 %) were far below toxic concentrations.

Generally, our results agree with previous observations by Vumma et al. (19), who found that nitrofurantoin did not affect the viability of 5637 (HTB-9) urinary bladder cells at concentrations of 30 and 300 μg/mL after 2, 4, and 12-hour exposure. Although both 5637 and T24 are bladder carcinoma cell lines, they primarily differ in their aggressiveness and genetic profiles. 5637 cells are derived from a less aggressive, non-invasive carcinoma, while T24 cells originate from a more aggressive, invasive carcinoma with a mutation in the H-Ras gene (20). Conversely, nitrofurantoin was reported to inhibit the growth of FANFT-induced murine bladder tumour (MBT2) and a human transitional cell carcinoma cell line (GIBB) in the concentration range of 30–120 μg/mL during an *in vitro* treatment lasting 2–9 days (21). T24 and MBT2 cells may yield different results due to species-specific differences in genetics, tumour induction, immune system interactions, and metabolic processes, with T24 being human-derived and MBT2 being murine. Also, the difference in results may be due to 6–25 times longer exposure times than those used in our study.

The two highest concentrations of hydroquinone tested in our study correspond to the highest bladder tissue exposure scenario from recommended treatment doses. Both significantly reduced survival and affected the morphology of T24 cells. The toxic effect was enhanced during cell recovery, which suggests that hydroquinone enters cells and initiates processes that continue during cell reproduction. Lee et al. (22) reported that 44 μg/mL of free hydroquinone induced acute toxicity within 4 h in human kidney proximal tubule epithelial cells (HK-2) and the transformed human liver epithelial (THLE-2) cell line. In contrast, our study revealed that concentrations of 20 and 50 μg/mL reduced survival only after 8 h in T24 cells, indicating higher resistance compared to normal cells and suggesting reversible damage from short (<8 h) exposures.

We found that hydroquinone started to exert cytotoxic and oxidative effects at ≥50 μg/mL in unrecovered T24 cells. Following the 24-hour cell recovery, the cytotoxic effect remained visible (Figure 1b), but the redox status recuperated and did not differ from the negative control. However, the highest concentrations of hydroquinone (corresponding to consuming one cup of bearberry leaf tea or a daily consumption (320 or 900 μg/mL, respectively) exhibited cytotoxic and oxidative effects regardless of cell recovery. It is possible that hydroquinone-induced disruption of T24 cell membrane integrity caused irreparable damage unsuitable for ROS measurement. These concentrations caused permanent cell damage, as T24 cell survival decreased to ~30 %, regardless of exposure time. However, at lower concentrations over 8 h, reversible damage occurred with no lasting effects after recovery.

The combined effects of nitrofurantoin and hydroquinone were significant in unrecovered T24 cells at all concentrations, affecting both the survivability and oxidative status (Figure 1, panel c1), while in the recovered cells, these effects subsided only at the lowest hydroquinone concentrations (Figure 1, panel c2). Table 2 shows a synergistic effect of the combination on survival in recovered cells and on oxidative status in unrecovered cells. However, the very low *β*_12_ factor values raise doubt if these compounds interacted synergistically in all mixture combinations with different concentrations and ratios.

**Table 2:** Benchmark dose (BMD) and confidence intervals BMDI (BMDL-BMDU) for hydroquinone plus nitrofurantoin mixtures and results of factorial regression analyses to determine their effect on T24 cell survival and induction of ROS after 8 h exposure

**Treatment**	**Response**	**PROAST software** ^#^				**STATISTICA software** ^*^			**Activity**	
		**BMD**	**BMDL (μg/mL)**	**BMDU**	**Effect**	**Multiple R (model testing)**	**P-value**	*β*_12_	**P-value**	
Unrecovered cells	% of survival	10.64	5.76	15.2	**yes**	0.64	<0.01	+2.21×10^−5^	0.11	/
Recovered cells (after 24 h)	% of survival	33.99	21.6	49	**yes**	0.72	<0.01	+9.38×10^−5^	**<0.01**	**synergistic**
Unrecovered cells	ROS induction	16.04	13.6	21.2	**yes**	0.53	<0.01	+0.0001	**<0.01**	**synergistic**
Recovered cells (after 24 h)	ROS induction	15.4	0.532	227	no	0.62	<0.01	−1.55×10^−5^	0.48	/

# BMD – benchmark dose; BMDL – lower 95 % confidence limit of the benchmark dose; BMDU – upper 95 % confidence limit of the benchmark dose; confirmation of the dose-response effect: BMD/BMDL <20 and BMDU/BMDL <50.

* Factorial regression model: positive (+) β12 factor – synergistic effect/activity, negative (−) β12 factor – antagonistic effect/activity (only if P<0.05)

### Genome instability effects

The CBMN Cyt assay used in this study to elucidate the toxic effects of hydroquinone and nitrofurantoin provides an integrated assessment of multiple cellular endpoints, including DNA and chromosomal damage, as well as disturbances in intracellular proteins involved in mitotic spindle assembly and cytoskeletal organisation (23). Previous studies have suggested that hydroquinone may disrupt intracellular protein components, thereby contributing to cellular dysfunction. Aspengren et al. (24) have shown that hydroquinone (1.1–5.5 μg/mL) disrupts the dermal melanophores of *Xenopus laevis* by reorganising microtubules. Oxidative stress alters the cytoskeleton, and maintaining this balance is crucial for normal cellular function. In a study by Kim et al. (25), the high hydroquinone concentration of 50 μmol/L (corresponding to 5.51 μg/mL) increased apoptosis of human lymphoblastic leukaemia cells (Jurkat cells) via ERK (contributes to cell growth while inhibiting apoptosis) and caspase inhibition, while the lower concentration of 5 μmol/L (0.55 μg/mL) increased the proportion of cells in the S phase of the cell cycle, possibly due to effects on microtubule distribution.

We found that hydroquinone (20 and 50 μg/mL) negatively affected the adhesion of T24 cells on glass slides 2 h after treatment, which made it impossible to analyse them. Moreover, nitrofurantoin slowed down T24 cell division, resulting in insufficient binucleated cells in the treated samples. Consequently, a follow-up experiment was carried out for both 2 and 8 h using lower doses of hydroquinone alone (5 and 10 μg/mL) as well as a combination of hydroquinone and nitrofurantoin for 2 h (5 or 10 μg/mL combined with 167 μg/mL).

The results of the CBMN Cyt assay with the lowered hydroquinone concentrations (5 and 10 μg/mL) are presented in Table 3. The MN count was significantly higher in all treated samples at both exposure times compared to the negative control. The highest count was observed in the sample treated with the mixture of hydroquinone (5 μg/mL) and nitrofurantoin (167 μg/mL) for 2 h (MIX 1) and was significantly higher than the sum of MNs in samples treated with individual compounds (P=0.001; chi-squared test), which we consider a synergism because the effect of the mixture is greater than the sum of individual compounds.

**Table 3:** Formation of micronuclei (MN), nucleoplasmic bridges (NPB), and nuclear buds (NB) in T24 cells after treatment with nitrofurantoin (N), hydroquinone (HQ), and their mixture over 2 and 8 h

**Sample**	**Time**	**Micronuclei**							**Nucleoplasmic bridges**		**Nuclear buds**	
		**Total (MN)_6000_ ± SD**	**Mean (MN)_1000_ ± SD**	**Total (BN_MN_)_6000_ ± SD**	**Mean (BN_MN_)_1000_ ± SD**	**Distribution of BN_MN_ cells with**						
						**1 MN**	**2 MN**	**3 MN**	**Mean (NPB)_1000_ ± SD**	**Total (NPB)_6000_ ± SD**	**Mean (NB)_1000_ ± SD**	**Total (NB)_6000_ ± SD**
NC_1_	2 h	86	14.3±0.52	78	13±1.05	71	6	1	1.2±0.75	7	1.3±0.75	8
NC_2_		75	12.5±1.03	69	11.5±0.53	63	6	0	1.5±0.84	9	0.7±0.51	4
N (167 μg/mL)		↑135^e^	22.5±3.99	↑127	21.2±4.36	118	7	1	9±2.53	↑54^d,e^	5±1.55	↑30^e^
HQ (5 μg/mL)		↑125^e^	20.8±4.02	↑110^e^	18.3±2.94	96	13	1	10.3±1.51	↑62^d^	5.5±1.52	↑33^e^
HQ (10 μg/mL)		↑145^e^	24.2±2.93	↑135	22.5±2.26	125	10	0	18.5±3.39	↑111^a,b,c^	6.2±1.47	↑37^a^
MIX 1		↑187^a,b,c,d^	31.2±5.15	↑165^a,c^	27.5±4.46	141	23	0	14.2±4.79	↑85^a,b^	8.5±2.2	↑51^a,b^
MIX 2		/	/	/	/	/	/	/	/	/	/	/
PC		↑138^e^	23 3±3.33	↑107^e^	17.8±2.21	17	17	4	7.3±2.1	↑44^d,e^	3.5±0.92	↑21^d^
NC_1_	8 h	72	12 1±.09	68	11.3±1.37	6	6	0	1.2±0.41	7	0.8±0.05	5
NC_2_		65	10.8±1.47	62	10.3±1.51	2	2	0	1±0.15	6	0.7±0.03	4
HQ (5 μg/mL)		↑167^d^	27.8±2.99	↑143^d^	23.8±1.17	18	18	3	30.2±2.08	↑46^a^	5.5±1.05	↑33^a,c^
HQ (10 μg/mL)*		↑133^a,c*^	22.2±3.65	↑112^a,c*^	18.7±2.73	13	13	4	19±1.03	↑19^a*^	7.3±1.97	↑44^a,b*^
PC		↑147^d^	24.5±3.99	↑133^d^	22.2±3.13	14	14	0	11.8±3.06	↑71^b,c^	8.5±1.16	↑51^b,c^

NC_1_ – negative control, non-treated cells; N – nitrofurantoin; HQ – hydroquinone; MIX 1 – mixture of N (167 μg/mL) and HQ (5 μg/mL); MIX 2 – mixture of N (167 μg/mL) and HQ (10 μg/mL); PC – positive control (0.4 μg/mL); NC_2_ – negative control, cells treated with 0.16 % DMSO used for stock of nitrofurantoin preparation.

* Expressed on a total of 3000 binucleated cells or 500 per sample. ↑ significantly higher than NC_1_ (P<0.05; chi-squared test). Different superscript letters denote significant difference between groups (P<0.05; chi-squared test):

a – from PC,

b - from N,

c – from HQ (5 μg/mL),

d – from HQ (10 μg/mL),

e – from MIX 1

In the sample treated with the mixture of 10 μg/mL hydroquinone with nitrofurantoin (MIX 2) for 2 h, hydroquinone had a significant effect on the adhesion of T24 cells, rendering cell scoring impossible (Table 3). Furthermore, due to the insufficient number of binucleated cells, the results for hydroquinone (10 μg/mL) are expressed for total of 3000 (instead of 6000) binucleated T24 cells.

Exposure duration did not affect the MN counts in the samples treated with hydroquinone (Table 3).

The occurrence of NBs in untreated control binucleated T24 cells was quite low (1.3±0.75 per 1000 cells). Treating T24 bladder cancer cells with nitrofurantoin and hydroquinone, as well as their combination for 2 h led to a significant increase in NBs compared to untreated control. The highest NB frequency was observed in the sample treated with MIX 1, which is significantly higher than in samples treated with individual compounds (P=0.036; chi-squared test), indicating a synergistic effect. NB frequency in other samples ranged from 5.0±1.55 to 8.5±2.2 per 1000 binucleated cells (Table 3).

Jurica et al. (26) observed no impact on the formation of micronuclei in lymphocytes after a 24-hour treatment with the 8 μg/mL concentration of hydroquinone. However, lymphocytes had statistically significantly more NBs compared to the negative control, while the concentrations of 140 and 280 μg/mL completely blocked lymphocyte division due to nuclear budding occurring during the S-phase of the cell cycle. It is believed that DNA repair results in excessive formation of nuclear buds, concentrated in the peripheral part of the nucleus (27). The differences between from our findings may therefore be due to differences in cell lines used for research.

NPB frequency in all treated cells was significantly higher than in untreated control, and the highest was recorded for 10 μg/mL hydroquinone alone (Table 3). It was also significantly higher than in T24 cells treated with 5 μg/mL hydroquinone alone, which was not the case with MN and NB.

MIX 1 yielded lower NPB frequency than 10 μg/mL hydroquinone alone, but the effect was significantly higher than with nitrofurantoin alone. Results obtained for MIX 1 suggest that nitrofurantoin and hydroquinone in the mixture act synergistically (P=0.022, chi-squared test).

It was not possible to determine the number of NB and NPB in the sample treated with a mixture of nitrofurantoin (167 μg/mL) and hydroquinone (10 μg/mL; MIX 2) for 2 h due to an insufficient number of binucleated cells on the slide. The 10 μg/mL hydroquinone significantly affected the adhesion of T24 cells, so the results are expressed per 500 binucleated cells instead of 3000. The number of NB is statistically significantly higher in the sample treated with a concentration of hydroquinone of 10 μg/mL for 8 h compared to the lower concentration, while there is no statistically significant difference in the number of NPB.

Exposure duration (2 and 8 h) did not significantly affect the number of NB and NPB in the samples treated with hydroquinone.

Table 4 shows significantly lower nuclear division index (NDI) in the cells exposed for 2 h to both nitrofurantoin concentrations and to both mixtures. Furthermore, mixture NDI was significantly lower than with either compound alone. Considering that hydroquinone treatment did not significantly lower the NDI, our results suggest that it potentiates the negative effect of nitrofurantoin in the mixture.

**Table 4:** Cell viability, cytostatic effect, and parameters of cell proliferation in T24 cells after treatment with nitrofurantoin, hydroquinone, and their mixture over 2 and 8 h

**Sample**	**Time**	**AP (%)**	**Cytostatic effect**		**Parameters of cell proliferation**	
			**CBPI**	**% cytostasis**	**NDI**	**RI (%)**
NC	2 h	0.13	1.718	0	1.726	100
N (167 μg/mL)		0.17	↓1.445	27.3	↓1.447	61.94
N (330 μg/mL)		0.18	↓1.259	45.9	↓1.261	36.94
HQ (5 μg/mL)		0.15	1.695	2.3	1.699	96.82
HQ (10 μg/mL)		0.25	1.666	5.2	1.671	92.89
MIX 1		0.13	↓1.225	49.3	↓1.225	31.28
MIX 2		0.15	↓1.077	64.1	↓1.077	10.68
NC	8 h	0.17	1.558	0	1.561	100
N (167 μg/mL)		0.25	↓1.058	50.0	↓1.059	10.29
HQ (5 μg/mL)		0.28	1.449	10.9	1.453	80.51
HQ (10 μg/mL)		↑0.83^*^	↓1.229	32.9	↓1.231	40.96

AP – number of cells in apoptosis (%); CBPI – Cytokinesis-block proliferation index; HQ – hydroquinone; MIX 1 – mixture of N (167 μg/mL) and HQ (5 μg/mL); MIX 2 – mixture of N (167 μg/mL) and HQ (10 μg/mL); N – nitrofurantoin; NC – negative control; NDI – nuclear division index; RI – replication index. ↓ significantly lower than NC (P<0.05; chi-squared test).

* Expressed on a total of 3000 cells

The 8 h exposure to nitrofurantoin and hydroquinone had an even stronger effect. All the above also applies to the value of the replication index (RI, Table 4). Compared to negative control, the only significant rise in apoptotic T24 cells was determined in the group treated with 10 μg/mL hydroquinone for 8 h (25/3000) (P<0.05, chi-squared test).

The cytostatic effects, cytokinesis-block proliferation index (CBPI) in particular, completely reflect NDI findings and are dose-dependent. Significantly higher percentage of cytostasis caused by both mixtures in T24 cells after 2 h of exposure compared to nitrofurantoin alone suggests that hydroquinone in the mixture potentiates the cytostatic activity of nitrofurantoin.

Cell kinetics findings suggest that nitrofurantoin inhibits or halts the growth and division of T24 cells. This effect is dose-dependent and increases with longer treatment time. One study (28) confirmed the genotoxic potential of nitrofurantoin in young and adult mice *in vivo*, showing that young mice (3 weeks old) were more sensitive to its genotoxic effects than adult mice (8 weeks old) and that response in young mice lasted longer. The application of the CBMN test *in vivo* revealed a statistically significantly higher number of micronuclei in reticulocytes from mice treated with 5, 10, and 50 mg/kg nitrofurantoin compared to the control group.

Additionally, previous study (26) demonstrated a significant disruption in lymphocyte kinetics by hydroquinone at a concentration of 8 μg/mL, which supports this study's findings regarding its impact on chromosomal aberrations and abnormal mitotic processes, including MN formation and sister chromatid alterations without direct evidence of mutagenicity.

### Protein analysis

Table 5 shows proteins whose expression changed significantly in T24 cells treated for 8 h v untreated cells, as identified with the permutation test. In total, 15 differentially expressed proteins (DEPs) were identified after nitrofurantoin treatment, 28 after hydroquinone treatment, and 44 after treatment with their mixture (167 μg/mL nitrofurantoin and 20 μg/mL hydroquinone).

**Table 5:** Proteomic analysis results obtained after treatment with nitrofurantoin, hydroquinone, and their mixture over 8 h (fold change cut-off ≥1.5 and ≤0.667)

**Condition**	**Protein name^a^**	**Accession number^b^**	**Alternate ID^c^**	**Permutation test (P-value)**	**Fold change^d^**
Hydroquinone	Albumin	A0A0C4DGB6	ALB	< 0.0001	0.3
	Guanine nucleotide-binding protein G(s) subunit alpha isoforms short	A0A7I2V5R6	GNAS	0.001	0.4
	Sideroflexin-1	Q9H9B4	SFXN1	0.004	0.5
	THO complex subunit 4	E9PB61	ALYREF	< 0.0001	0.4
	CD44 antigen	P16070	CD44	< 0.0001	0.5
	Tropomyosin alpha-3 chain	A0A087WWU8	TPM3	0.003	0.5
	Zinc transporter ZIP3	F5H385	SLC39A3	0.003	0.3
	Heterogeneous nuclear ribonucleoprotein U	A0A1W2PPS1	HNRNPU	0.002	0.5
	Cathepsin B	A0A7P0NGZ6	CTSB	0.002	0.5
	Succinate–CoA ligase [ADP/GDP–forming] subunit alpha, mitochondrial	P53597	SUCLG1	0.001	0.6
	Non-histone chromosomal protein HMG–17	P05204	HMGN2	0.003	0.55
	Procollagen–lysine,2–oxoglutarate 5–dioxygenase 2	O00469	PLOD2	< 0.0001	1.5
	Synaptic vesicle membrane protein VAT–1 homolog	Q99536	VAT1	0.003	1.7
	Filamin-C OS=*Homo sapiens*	Q14315	FLNC	0.0009	1.7
	Mesencephalic astrocyte-derived neurotrophic factor	P55145	MANF	0.004	1.7
	Tyrosine-protein phosphatase non–receptor type 1	P18031	PTPN1	0.0003	2.3
	Delta–1–pyrroline–5–carboxylate synthase	P54886	ALDH18A1	< 0.0001	1.9
	UDP–glucose:glycoprotein glucosyltransferase 1	Q9NYU2	UGGT1	< 0.0001	2.0
	Histone H1.4	P10412	H1–4	0.002	2.4
	Lysophospholipid acyltransferase 7	Q96N66	MBOAT7	0.001	4.2
	ATP-dependent 6–phosphofructokinase, platelet type	Q01813	PFKP	0.001	4.4
	LIM domain only protein 7	F8WD26	LMO7	0.001	3.3
	MARCKS–related protein	P49006	MARCKSL1	0.002	8.1
	Aminoacyl–tRNA hydrolase	J3KQ48	PTRH2	0.0001	5.6
	A–kinase anchor protein 12	Q02952	AKAP12	< 0.0001	Unique
	Fascin	Q16658	FSCN1	< 0.0001	Unique
	ATP–dependent RNA helicase DDX24	Q9GZR7	DDX24	0.0002	Unique
	Aladin	Q9NRG9	AAAS	0.001	Unique
Nitrofurantoin	Probable 28S rRNA (cytosine(4447)–C(5))-methyltransferase	P46087	NOP2	0.002	9.5
	ATPase family AAA domain–containing protein 3A	Q9NVI7	ATAD3A	0.0003	4.5
	Proteasome subunit beta type–3	P49720	PSMB3	0.002	4.8
	H/ACA ribonucleoprotein complex subunit DKC1	O60832	DKC1	0.002	4.9
	40S ribosomal protein S15	K7ELC2	RPS15	0.0004	2.6
	Integrin alpha–6	A0A8C8KBL6	ITGA6	< 0.0001	5.5
	Serine/arginine–rich splicing factor 1	J3KTL2	SRSF1	0.001	3.7
	Caveolae–associated protein 3	E9PIE3	CAVIN3	0.002	6.4
	Membrane–associated progesterone receptor component 1	O00264	PGRMC1	0.001	9.3
	WD40 repeat-containing protein SMU1	Q2TAY7	SMU1	0.0003	16.2
	Oxygen–dependent coproporphyrinogen-III oxidase, mitochondrial	P36551	CPOX	0.004	42.3
	MARCKS–related protein	P49006	MARCKSL1	0.002	11.6
	Aminoacyl–tRNA hydrolase	J3KQ48	PTRH2	0.0001	16.1
	Synaptic vesicle membrane protein VAT–1 homolog	Q99536	VAT1	0.003	6.8
	Sec1 family domain–containing protein 1	A0A7I2V3G4	SCFD1	0.0001	35.0
MIX	Integrator complex subunit 1	Q8N201	INTS1	0.0003	0.2
	Proteasome subunit beta type–3	P49720	PSMB3	0.002	0.4
	Protein disulfide-isomerase TMX3	Q96JJ7	TMX3	0.004	0.4
	Very–long–chain (3R)–3–hydroxyacyl–CoA dehydratase 2	Q6Y1H2	HACD2	< 0.0001	0.5
	Cathepsin B	A0A7P0NGZ6	CTSB	0.002	0.5
	Guanine nucleotide–binding protein G(s) subunit alpha isoforms short	A0A7I2V5R6	GNAS	0.001	0.4
	Albumin	A0A0C4DGB6	ALB	< 0.0001	0.3
	Stomatin–like protein 2, mitochondrial	Q9UJZ1	STOML2	0.001	0.5
	PC4 and SFRS1–interacting protein	O75475	PSIP1	0.001	0.6
	60S acidic ribosomal protein P0	P05388	RPLP0	< 0.0001	0.5
	Sideroflexin–1	Q9H9B4	SFXN1	0.004	0.4
	UDP–glucose:glycoprotein glucosyltransferase 1	Q9NYU2	UGGT1	< 0.0001	2.7
	Protein disulfide–isomerase A4	P13667	PDIA4	< 0.0001	1.9
	Endoplasmin	P14625	HSP90B1	< 0.0001	1.8
	Delta–1–pyrroline–5–carboxylate synthase	P54886	ALDH18A1	< 0.0001	1.7
	Calreticulin	P27797	CALR	< 0.0001	1.6
	Protein disulfide–isomerase A3	A0A8I5KT88	PDIA3	< 0.0001	1.6
	Heterogeneous nuclear ribonucleoprotein R	O43390	HNRNPR	< 0.0001	1.7
	Glutaminase kidney isoform, mitochondrial	O94925	GLS	< 0.0001	1.9
	Histone H1.5	P16401	H1–5	0.004	1.9
	Thioredoxin domain–containing protein 5	Q8NBS9	TXNDC5	< 0.0001	1.8
	Coactosin–like protein	Q14019	COTL1	0.001	2.1
	T–complex protein 1 subunit epsilon	P48643	CCT5	0.001	2.0
	Lysophospholipid acyltransferase 7	Q96N66	MBOAT7	0.001	1.9
	Splicing factor U2AF 65 kDa subunit	P26368	U2AF2	0.002	2.3
	Lamin–B1	P20700	LMNB1	< 0.0001	2.0
	LIM domain and actin–binding protein 1	Q9UHB6	LIMA1	0.0004	2.2
	CD44 antigen	P16070	CD44	< 0.0001	2.1
	Tyrosine–protein phosphatase non–receptor type 1	P18031	PTPN1	0.0003	1.8
	Mitochondrial import receptor subunit TOM70	O94826	TOMM70	0.001	3.2
	Histone H1.4	P10412	H1–4	0.002	3.4
	Thymosin beta–10	P63313	TMSB10	0.003	2.7
	Aminoacyl–tRNA hydrolase	J3KQ48	PTRH2	0.0001	4.9
	Brain acid soluble protein 1	P80723	BASP1	0.001	3.0
	Very–long–chain 3–oxoacyl–CoA reductase	Q53GQ0	HSD17B12	0.0001	3.1
	MARCKS–related protein	P49006	MARCKSL1	0.002	4.6
	ATP–dependent 6–phosphofructokinase, platelet type	Q01813	PFKP	0.001	4.8
	Podocalyxin	O00592	PODXL	< 0.0001	Unique
	Ras–related protein Ral–A	P11233	RALA	< 0.0001	Unique
	F–actin–capping protein subunit beta	B1AK88	CAPZB	< 0.0001	Unique
	CCN family member 1	O00622	CCN1	0.0001	Unique
	ATP–dependent RNA helicase DDX24	Q9GZR7	DDX24	0.0002	Unique
	Purine nucleoside phosphorylase	P00491	PNP	0.001	Unique
	3–ketoacyl–CoA thiolase, peroxisomal	P09110	ACAA1	0.001	Unique

a – protein name in the database (UniProt);

b – accession number in the database (UniProt);

c – denotes gene name.

d – ratio of average signal values of reporter ions treatment / control sample;

Unique – protein identified in the treated samples but not in control

**Figure 2:**
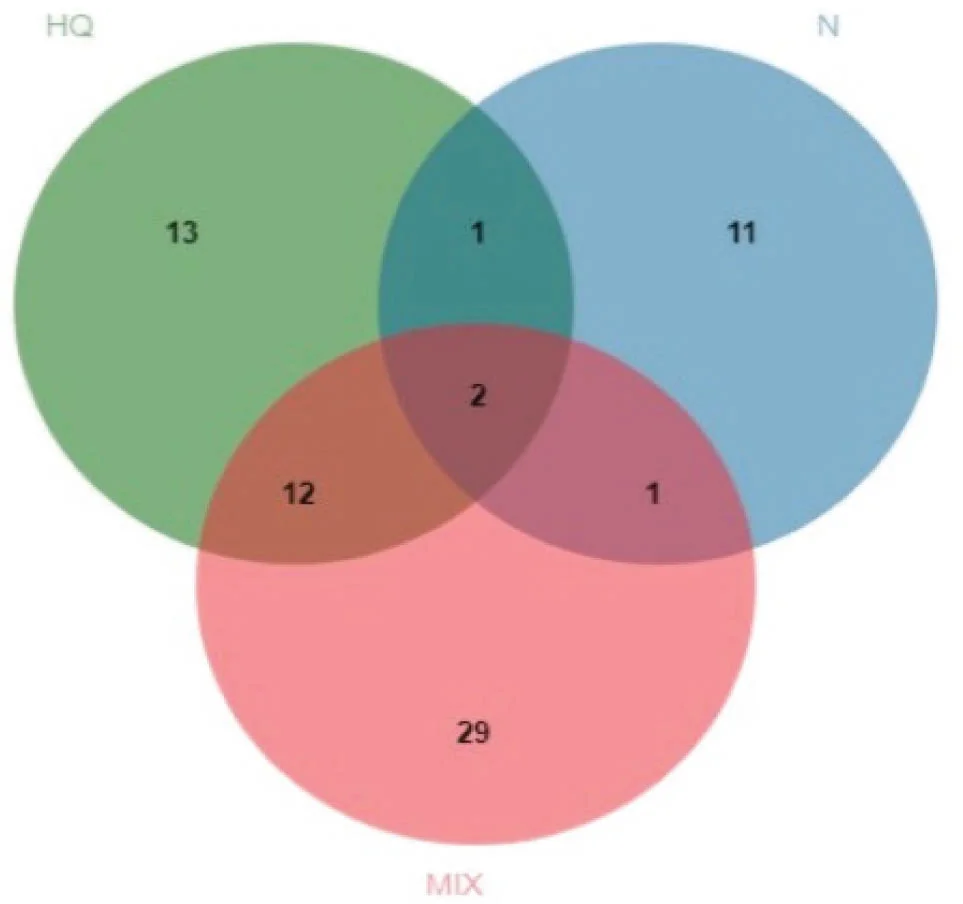
Venn diagrams comparing differentially expressed proteins obtained after treatment with nitrofurantoin (N), hydroquinone (HQ), and their mixture (MIX) after 8 h

The Venn diagram (Figure 2) shows that only two DEPs overlap between all conditions (MARCKSL1 and PTRH2), 12 between the mixture and hydroquinone (CTSB, GNAS, ALB, SFXN1, UGGT1, ALDH18A1, MBOAT7, CD44, PTPN1, H1-4, PFKP, and DDX24), one between nitrofurantoin and the mixture (PSMB3), and one between nitrofurantoin and hydroquinone (VAT1). Furthermore, 29 DEPs belong exclusively to the MIX group (INTS1, TMX3, HACD2, STOML2, PSIP1, RPLP0, PDIA4, HSP90B1, CALR, PDIA3, HNRNPR, GLS, H1-5, TXNDC5, COTL1, CCT5, U2AF2, LMNB1, LIMA1, TOMM70, TMSB10, BASP1, HSD17B12, PODXL, RALA, CAPZB, CCN1, PNP, and ACAA1), 11 to the nitrofurantoin alone group (NOP2, ATAD3A, DKC1, RPS15, ITGA6, SRSF1, CAVIN3, PGRMC1, SMU1, CPOX, and SCFD1), and 13 to the hydroquinone alone group (ALYREF, TPM3, SLC39A3, HNRNPU, SUCLG1, HMGN2, PLOD2, FLNC, MANF, LMO7, AKAP12, FSCN1, and AAAS).

The complete Gene Ontology (GO) database classification of DEPs (Table 6) shows that the mixture significantly affected the expression of proteins related to glutamine catabolism (GO:0006543), glutamate biosynthesis (GO:0006537), glutamine family amino acid biosynthesis (GO:0009084), cellular biosynthesis (GO:0044249), protein folding in endoplasmic reticulum (GO:0034975), protein folding (GO:0006457), protein maturation (GO:0051604), and response to endoplasmic reticulum stress (GO:0034976).

**Table 6:** Complete Gene Ontology (GO) classification of differentially expressed proteins (DEPs) in the cells after treatment with the mixture of nitrofurantoin and the hydroquinone by biological processes

**GO biological process**	No. of proteins in reference list^*^	**Genes encoding differentially expressed proteins**	**P-value**	**False discovery rate**
Glutamine catabolic process (GO:0006543)	3	GLS	1.22×10^−5^	3.07×10^−2^
Glutamate biosynthetic process (GO:0006537)	4	GLS	2.43×10^−5^	4.60×10^−2^
Cellular biosynthetic process (GO:0044249)	3530	CALR, UGGT1, GLS, ALDH18A1, INTS1, U2AF2, HSP90B1, TXNDC5, PDIA4, PDIA3, STOML2, HSD17B12, CCT5, PSIP1, RPLP0, MBOAT7, HACD2, HNRNPR	2.13×10^−5^	9.75×10^−3^
Glutamine family amino acid biosynthetic process (GO:0009084)	15	GLS, ALDH18A1	3.54×10^−6^	1.78×10^−2^
Protein folding in endoplasmic reticulum (GO:0034975)	11	CALR, HSP90B1, PDIA3	1.29×10^−6^	4.72×10^−3^
Protein folding (GO:0006457)	223	CALR, UGGT1, HSP90B1, TXNDC5, PDIA4, PDIA3, CCT5	3.12×10^−7^	1.87×10^−2^
Protein maturation (GO:0051604)	494	CALR, UGGT1, HSP90B1, TXNDC5, PDIA4, PDIA3, STOML2, CCT6	6.00×10^−6^	2.27×10^−2^
Response to endoplasmic reticulum stress (GO:0034976)	226	CALR, UGGT1, HSP90B1, PDIA4, PDIA3, PTPN1	6.19× 10^−6^	4.59×10^−2^

* *Homo sapiens*, all genes in database were used as reference list, total No. 20580

Our proteomic analysis reveals the up-regulation of MARCKS-related protein and peptidyl-tRNA hydrolase 2 over 8 h. MARCKS-related protein controls cell movement by regulating actin cytoskeleton homeostasis and the formation of filopodia and lamellipodia. In its unphosphorylated form, it induces cell migration. When phosphorylated, it facilitates the formation and stabilisation of actin bundles, which, in turn, reduces actin plasticity and limits cell movement (29). Mitochondrial peptidyl-tRNA hydrolase 2 regulates the function of two transcriptional regulators, AES and TLE1, and through them promotes caspase-independent apoptosis (29).

In our previous study (8), we investigated proteome changes in human bladder T24 cells induced by hydroquinone after 2 h, and the results indicated increased expression of proteins related to the glycolysis (GO:0006096), response to stimulus (GO:0050896), and translational initiation (GO:0006413). In this study, we prolonged the treatment to 8 h and detected a completely changed, differentially expressed set of proteins (DEPs). Although gene ontology analysis did not classify the DEPs into specific biological processes, according to the 2023 Universal Protein Knowledgebase (28), fascin, filamin-C, and tropomyosin alpha-3 chain are involved in the actin-binding molecular function (GO:0003779). Fascin is involved in the organisation of actin filament bundles and the formation of microspikes, membrane ruffles, and stress fibres. It plays an important role in forming a diverse set of cell protrusions, such as filopodia, and in cell motility and migration. Fascin also mediates the reorganisation of the actin cytoskeleton. Filamin-C plays a central role in muscle cells, likely as a large actin-cross-linking protein. It may be involved in reorganising the actin cytoskeleton in response to signalling events and is essential for normal myogenesis and for maintaining the structural integrity of muscle fibres. Tropomyosin alpha-3 chain is an actin-binding protein implicated in stabilising cytoskeletal actin filaments. At the molecular level, hydroquinone caused changes in the T24 cytoskeleton, quite likely because of the activated cytotoxic mechanisms.

After nitrofurantoin treatment, gene ontology analysis did not classify the set of DEPs into specific biological processes. However, our results suggest that nitrofurantoin primarily affected proteins involved in RNA metabolism (GO:0016070) such as ribosomal protein S15, H/ACA ribonucleoprotein complex subunit DKC1, 28S rRNA (cytosine(4447)-C(5))-methyltransferase, serine/arginine-rich splicing factor 1, WD40 repeat–containing protein SMU1, and proteasome subunit beta type-3. Probable 28S rRNA (cytosine(4447)-C(5))-methyltransferase may play a role in regulating the cell cycle and the increased nucleolar activity associated with cell proliferation (29). H/ACA ribonucleoprotein complex subunit DKC1 is a protein that catalyses the pseudouridylation of rRNA but can also promote cell-to-cell and cell-to-substratum adhesion, increase cell proliferation, and lead to cytokeratin hyper-expression (29). Proteasome subunit beta type-3 plays a key role in maintaining protein homeostasis by removing misfolded or damaged protein, and WD40 repeat-containing protein SMU1 is essential for normal mitotic spindle assembly and mitosis (29).

### Synergistic activity of nitrofurantoin and hydroquinone in mixture

Our study shows that the combination of nitrofurantoin (167 μg/mL) and hydroquinone (5 μg/mL) had a synergistic genotoxic effect in T24 cells compared to the individual compounds. Moreover, hydroquinone enhanced the cytostatic action of nitrofurantoin, particularly when the exposure was prolonged to 8 h. This finding is relevant, as it indicates a potential interaction that may alter the antibiotic's effectiveness. In contrast, a previous study examined the antioxidant properties of *Rosmarinus officinalis*, *Cynara scolymus* L., and *Hedera helix* extracts during nitrofurantoin therapy, and reported that these plant extracts reduced the prooxidative impact (~50 %) of nitrofurantoin (30).

The up-regulation of proteins involved in biological processes such as the glutamine catabolism (GO:0006543), glutamate biosynthesis (GO:0006537), cellular biosynthesis (GO:0044249), and glutamine family amino acid biosynthesis (GO:0009084) strongly suggests that the synergistic activity of nitrofurantoin and hydroquinone activates this mechanism to prevent severe damage to biomolecules (31). The synergistic activity of the combination also activated biological processes such as protein folding in the endoplasmic reticulum (GO:0034975), protein folding (GO:0006457), protein maturation (GO:0051604), and response to endoplasmic reticulum stress (GO:0034976). The endoplasmic reticulum plays a crucial role in protein quality control and homeostasis, including degradation, protein chaperones, and autophagy. The accumulation of misfolded and unfolded proteins triggers stress in endoplasmic reticulum to restore proteostasis (32).

## CONCLUSION

This study demonstrates that a combination of nitrofurantoin and hydroquinone can have synergistic genotoxic and cytostatic effects on T24 bladder cells compared to each compound alone. Proteomic analysis indicates the activation of pathways involved in glutamine catabolism, glutamate synthesis, protein folding, and endoplasmic reticulum stress response, suggesting cellular defence mechanisms against proteotoxic stress. These findings also provide data that are relevant for human health risk assessment and potential regulatory evaluation of co-exposures involving herbal and pharmaceutical compounds. Nevertheless, these findings should be interpreted in light of the study's limitation of using a single bladder tumour cell line as the sole *in vitro* model. Future studies are warranted to validate the present findings in additional cell types, including non-tumour bladder cell lines and three-dimensional (3D) models of the urinary system, as well as in appropriate *in vivo* models.

## Abbreviations

ATCC: American Type Culture Collection
DCFH-DA: 2′,7′-dichlorofluorescein diacetate
DEP: differentially expressed protein
DMSO: dimethyl sulphoxide
GO: Gene Ontology (database)
HQ: hydroquinone
MIX: mixture of hydroquinone and nitrofurantoin
MN: micronuclei
N: nitrofurantoin
NB: nuclear buds
NDI: nuclear division index
NPB: nucleoplasmic bridges
ROS: reactive oxygen species
T24: human urinary bladder carcinoma cell line
TEAB: triethyl ammonium bicarbonate
TMT: tandem mass tag
